# Supplementary material for: CMash: fast, multi-resolution estimation of k-mer-based Jaccard and containment indices
Source: Bioinformatics. 2022 Jun 27;38(Suppl 1):i28–35. doi: 10.1093/bioinformatics/btac237 (PMC9235470; doi:10.1093/bioinformatics/btac237)
Supplement: btac237_Supplementary_Data [file btac237_supplementary_data.zip › btac237-suppl_data/Koslicki.70.sup.1.docx]

**Supplementary material**

# **Intuitive explanation for CMash workflow**

When utilizing CMash, the first step is to build a reference sketch for all the reference genomes. K-mers (with k=k_max) from each input genome will be scanned and hashed to generate sketches (Supplementary Figure S1a). These sketches will then be stored in a ternary search tree in which every distinct k-mer is represented by a root-to-leaf path. Then, k-mer truncation is achieved via prefix lookup and new sketches for a smaller k size can be generated in constant time on the fly (linear to the pre-defined sketch size) (Supplementary Figure S1b).

CMash is built upon the MinHash algorithm (Broder A Z., 1997). In contrast to most other MinHash-based tools (often implemented with a “bottom sketch” strategy) which utilize hash values instead of k-mers that hash to those values, CMash builds sketches from k-mer elements (Supplementary Figure S1a) as they enable the truncation-based estimation (Supplementary Figure S1b). With k-mer elements in a ternary search tree, we can arbitrarily truncate the sketch elements (k-mers) to any shorter length to obtain similarity estimation (such as Jaccard index or containment index). This truncation-based method, of which the efficiency is upper bounded by the sketch size, avoids the need to rebuild a sketch for other k values. In the main manuscript Figure 1d, we show an example of a ternary search tree to store the data while here in Supplementary Figure S1b we visualize the truncation-based method.


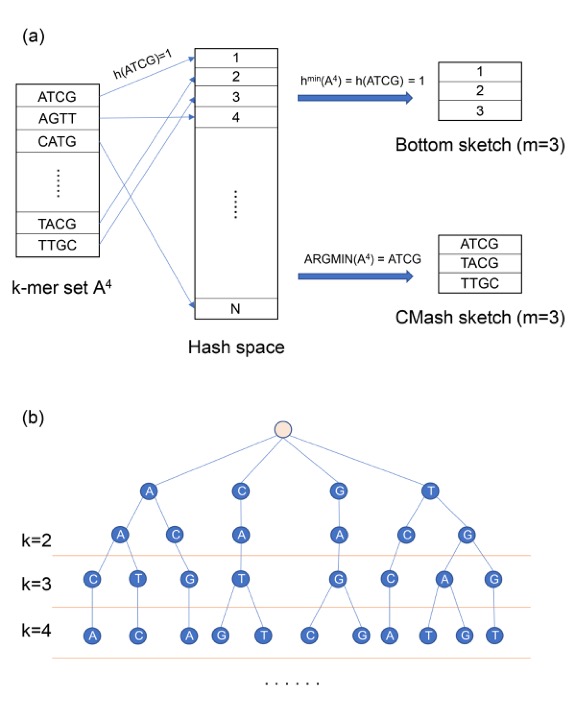


**Supplementary Figure S1: Intuitive data processing for CMash.** (a) Sketching method for CMash. Given some perfect hash function h (or breaking ties in some fashion if h is not a perfect hash function), k-mers can be randomly projected into some hash space, forming a random permutation from which we can build our sketch by hash values (bottom sketch) or by elements (CMash). (b) Conceptual k-mer storage and truncation. In CMash, we used a ternary search tree data structure to store the k-mer sketches (main manuscript Figure 1d) where each node in the trie was used to store a letter (identification information can be stored in leaf nodes), and empty edges in the trie are omitted. A prefix lookup effectively returns “truncated” k-mers of any shorter length.

**A motivation example for Cmash**

In many bioinformatics settings, such as in metagenomic analysis, the choice of k for a k-mer based analysis is frequently heuristic and relies on empirical results. For example, practitioners encounter this issue when performing k-mer based metagenomic taxonomic profiling (i.e. estimating the presence and relative abundances of taxa in a metagenomic sample) as well as when comparing similarity of sequences. In one such method, Mash, the authors demonstrated how the adjustment of containment cutoffs can affect the sensitivity and specificity (Ondov et al., 2019). Not only this, but our previous work demonstrated how k-mer size impacts sequence similarity which in turn correlates highly with evolutionary relatedness (Koslicki and Falush, 2016). Hence, the choice of k-mer size directly impacts at what granularity of evolutionary relatedness these k-mer based methods can distinguish. For example, in main manuscript Figure 2a, the pairwise JI values decrease at different rates, indicating sub-structure within the Brucella genus.

Adjusting k values gives us another dimension of freedom (besides the similarity cutoff) from which we can infer taxonomic boundaries and explore genomic composition. For example, large k-mer sizes can be used to identify sample sequences closely related to reference genomes, and subsequent progressively smaller k-mer sizes can be used to identify more divergent sequences in relationship to reference genomes.

In this motivation example, a randomly picked metagenomic sample (SRR1804890) from the Human Microbiome Project (Proctor, L. M., et al., 2019) was downloaded from SRA and then profiled by CMash to search for the presence of the 1000 random genomes described in section 3.2. Mash containment scores (Ondov et al., 2019) based on containment indices were arbitrarily selected to determine the status of present/absent of query genomes. Supplementary Figure S2 shows the number of detected genomes under different similarity cutoffs and k values. We can find that there are around 10 to 20 “core” genomes (and their close relatives) identified. More distal relationships can be captured when we relax k from 25 to 20. Tuning the similarity cutoffs is a common way to weigh the sensitivity and specificity, but we can quickly measure performance at many k-mer sizes simultaneously with only one pass of the data and significantly less storage space.


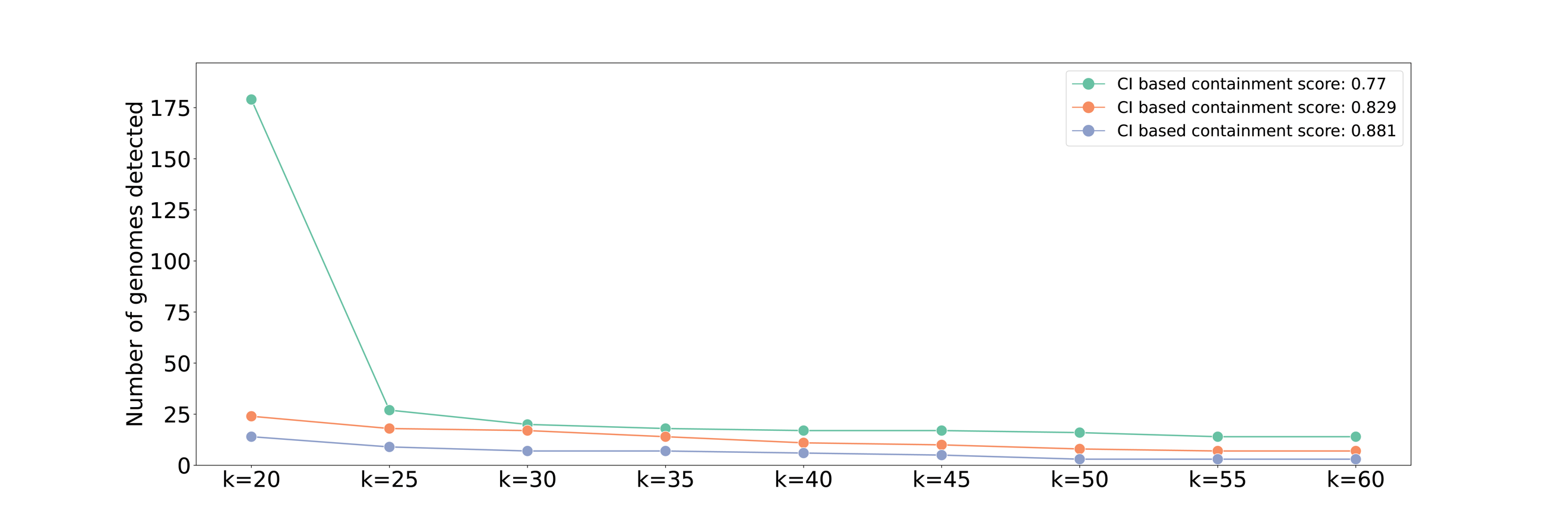


**Supplementary Figure S2. Using CMash to profile a human metagenomic sample**. The number of detected genomes depends on both the similarity cutoffs and the k values. Three containment score cutoffs are depicted corresponding to different choices of what containment score constitutes a reference genome as having strong evidence for appearing in the sample. The choice of k=20 or k=25 is crucial to identify more divergent genomes that are related to the reference genomes.

**Comparison of CMash to Sourmash and Mash**

Here we compared the performance of CMash to Sourmash and Mash utilizing the same data in section 3.1. Pairwise Jaccard indices between 30 Brucella genomes were estimated by CMash, Sourmash and Mash for k values ranging from 15 to 60 in steps of 5. The default settings were utilized for all tools except changing the sketch size of Sourmash and Mash from 1000 to 2000 to match the CMash setting. Mash only supports k values from 1 to 32, so the Mash results are only shown to k=30.

The results in Supplementary Figure S3 are similar to the results in main manuscript Figure 2. Though CMash takes advantage of a truncation-based method, all three tools are built on the bottom sketch strategy (note CMash utilizes elements instead of hash values) with a single hash function. The major differences are in computational perspective: Sourmash and Mash need to recompute the sketch when a new k-mer size is selected, in contrast to CMash. We can observe that the results from CMash, Sourmash, and Mash are close to the ground truth and they behave similarly regarding the deviation values as well as the relative errors. This is part of the reason why we compare CMash to a classic MinHash algorithm in the manuscript: tools based on the bottom sketch strategy of the classic MinHash algorithm are expected to have similar estimation performance theoretically.


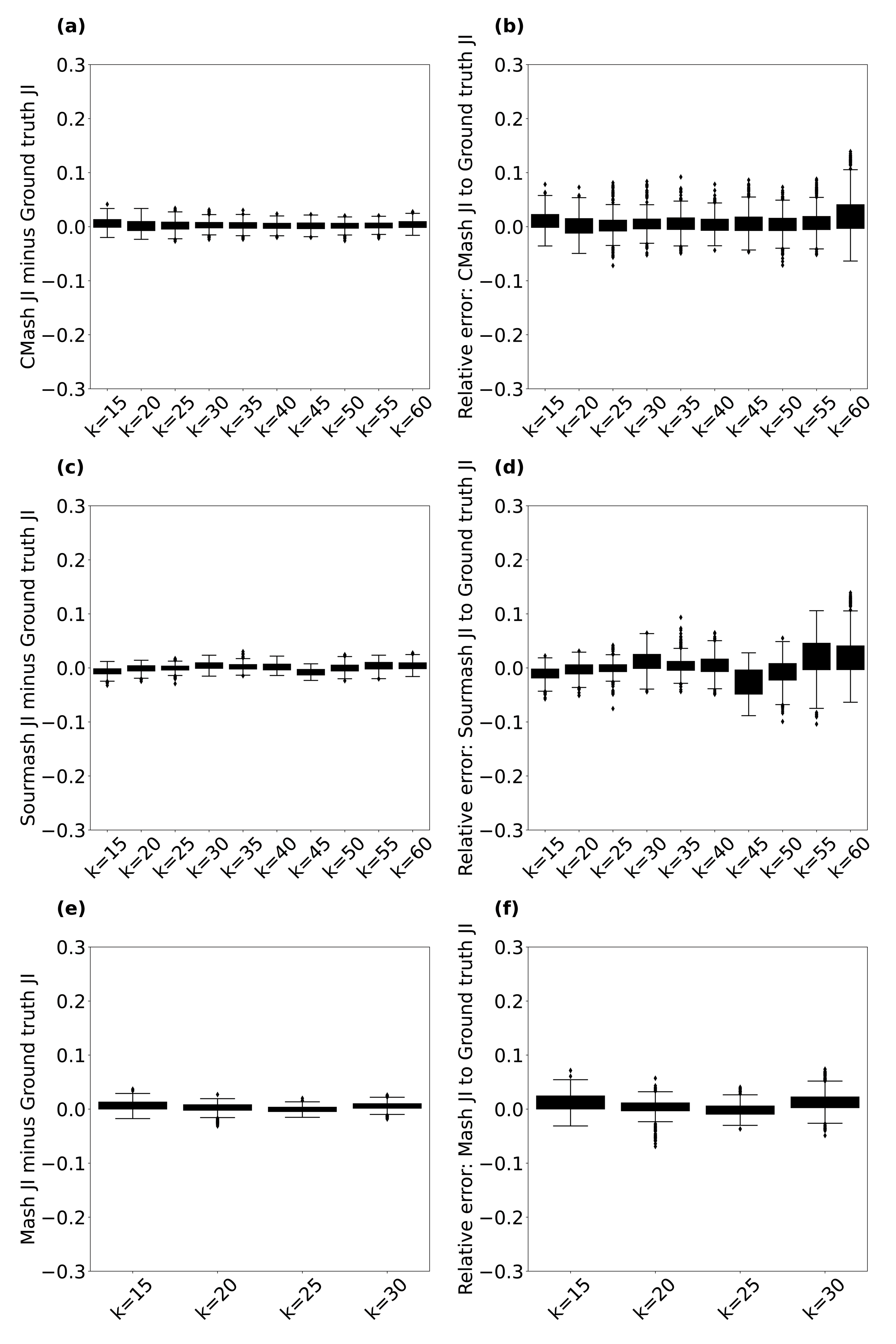


**Supplementary Figure S3. CMash results are comparable to Sourmash and Mash regarding JI estimation.** (a) Boxplot of JI value differences between CMash and the ground truth. (b) Boxplot of relative errors of CMash compared to the ground truth. (c) Boxplot of JI value differences between Sourmash and the ground truth. (d) Boxplot of relative errors of Sourmash compared to the ground truth. (e) Boxplot of JI value differences between Mash and the ground truth. (f) Boxplot of relative errors of Mash compared to the ground truth.

**Empirical distribution of the bias factor**

Here we compare the theoretically derived expected bias value with the actual bias value computed from the data. We analyzed both the observed as well as the expected bias factor for four groups of data across different JI ranges.

Hierarchical clustering was performed on the 1000 random genomes and four groups were manually selected with pairwise JI values at k=30 in different ranges: “near-zero” for values less than 0.01; “mid” for values between 0.3 and 0.6; “high” for values between 0.5 and 0.8; “near-one” for values between 0.9 and 1. The expected bias factors were calculated in a brute force manner: we enumerate all k-mers at the max k value and all k-mers at the truncated k value to calculate equation (18) in the main manuscript. On the contrary, the observed bias factors were derived from CMash estimation (estimated JI divided by the true JI). Supplementary Figure S4 shows the results for the distribution of the bias factor in different groups and by different truncation lengths (from max k value of 60).

Of note, the observed results in Supplementary Figure S4a might be overly optimistic because there are many 0s in both the estimation and ground truth, which causes issues when comparing ratios. Other than that, the trends observed in Supplementary Figure S4 b~d align well with our theoretical analysis in section 2.4: the expected bias factor will decrease when k value increases (i.e. fewer letters are truncated) or when similarity increases (i.e. higher JI value). The expected estimation biases in groups with JI of 0.3 or higher are close to 1, indicating a good theoretical bound for CMash estimates.

To our surprise, the observed estimation bias is much smaller and closer to 1. One possible explanation is that the containment MinHash method (Koslicki, D., & Zabeti, H. 2019; Ondov, Brian D., et al., 2019), which utilizes a streaming method to process all k-mers in one of the two genomes, may reduce the estimation deviation during truncation. More theoretical work is needed in the future to validate this hypothesis. In any case, given moderate truncation values and moderate to high genome similarity, CMash appears to have only slight biases in both theory and practice.


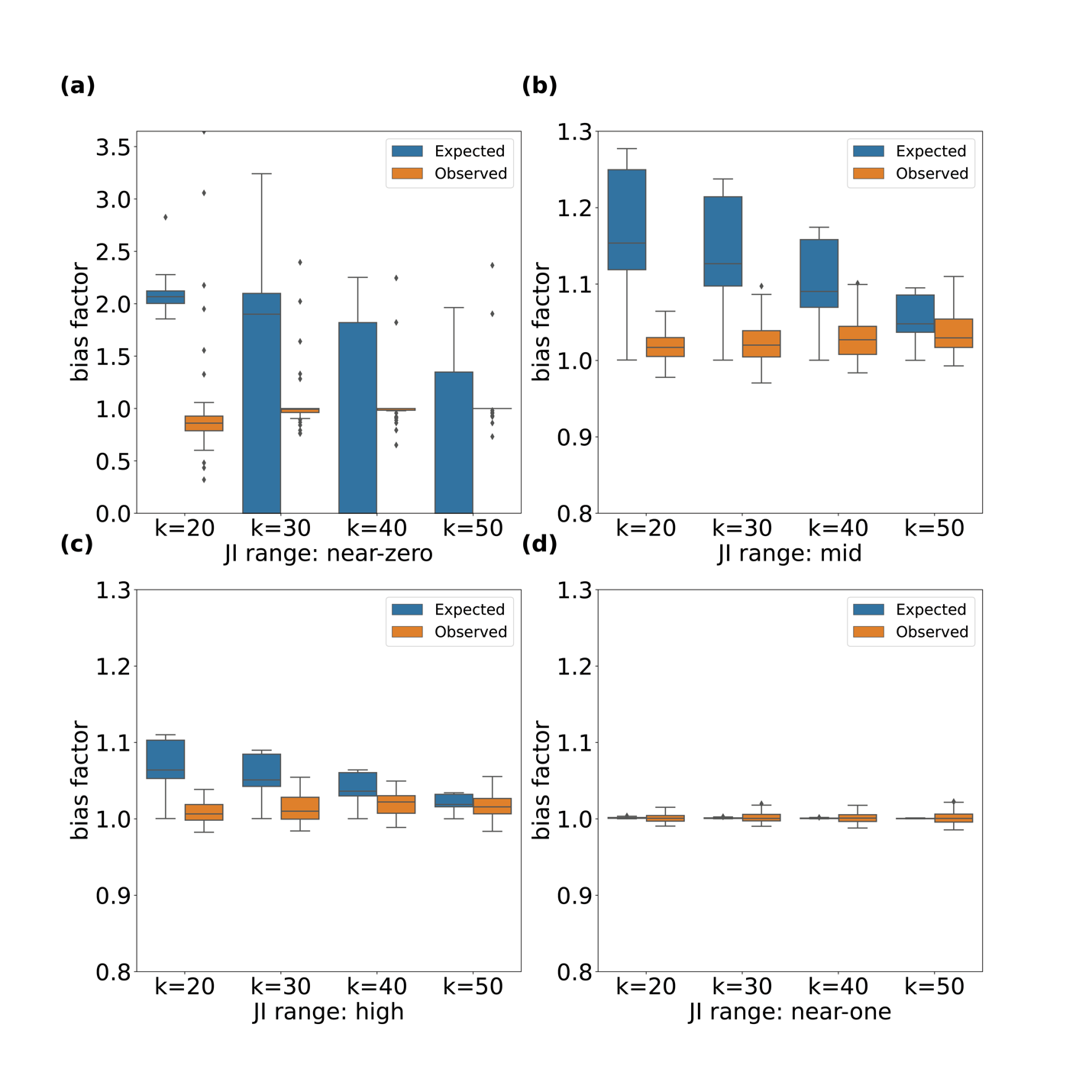


**Supplement Figure S4. Empirical distribution of the bias factor due to truncation.** The expected and observed estimation bias for groups with (a) JI values that are close to 0; (b) JI values ranging from 0.3 to 0.6; (c) JI values ranging from 0.5 to 0.8; and (d) JI values ranging from 0.9 to 1.

**Supplementary References**

1. Broder, Andrei Z. "On the resemblance and containment of documents." *Proceedings. Compression and Complexity of SEQUENCES 1997 (Cat. No. 97TB100171)*. IEEE, 1997.

2. Koslicki, David, and Daniel Falush. "MetaPalette: ak-mer Painting Approach for Metagenomic Taxonomic Profiling and Quantification of Novel Strain Variation." *MSystems* 1.3 (2016): e00020-16.

3. Koslicki, David, and Hooman Zabeti. "Improving minhash via the containment index with applications to metagenomic analysis." *Applied Mathematics and Computation* 354 (2019): 206-215.

4. Ondov, Brian D., et al. "Mash Screen: high-throughput sequence containment estimation for genome discovery." *Genome biology* 20.1 (2019): 1-13.

5. Proctor, L. M., et al. "The Integrative Human Microbiome Project." *Nature* 569.7758 (2019): 641-648.
