# Supplementary figures and images for: CMash: fast, multi-resolution estimation of k-mer-based Jaccard and containment indices
Source: Bioinformatics. 2022 Jun 27;38(Suppl 1):i28–35. doi: 10.1093/bioinformatics/btac237 (PMC9235470; doi:10.1093/bioinformatics/btac237)

(a)

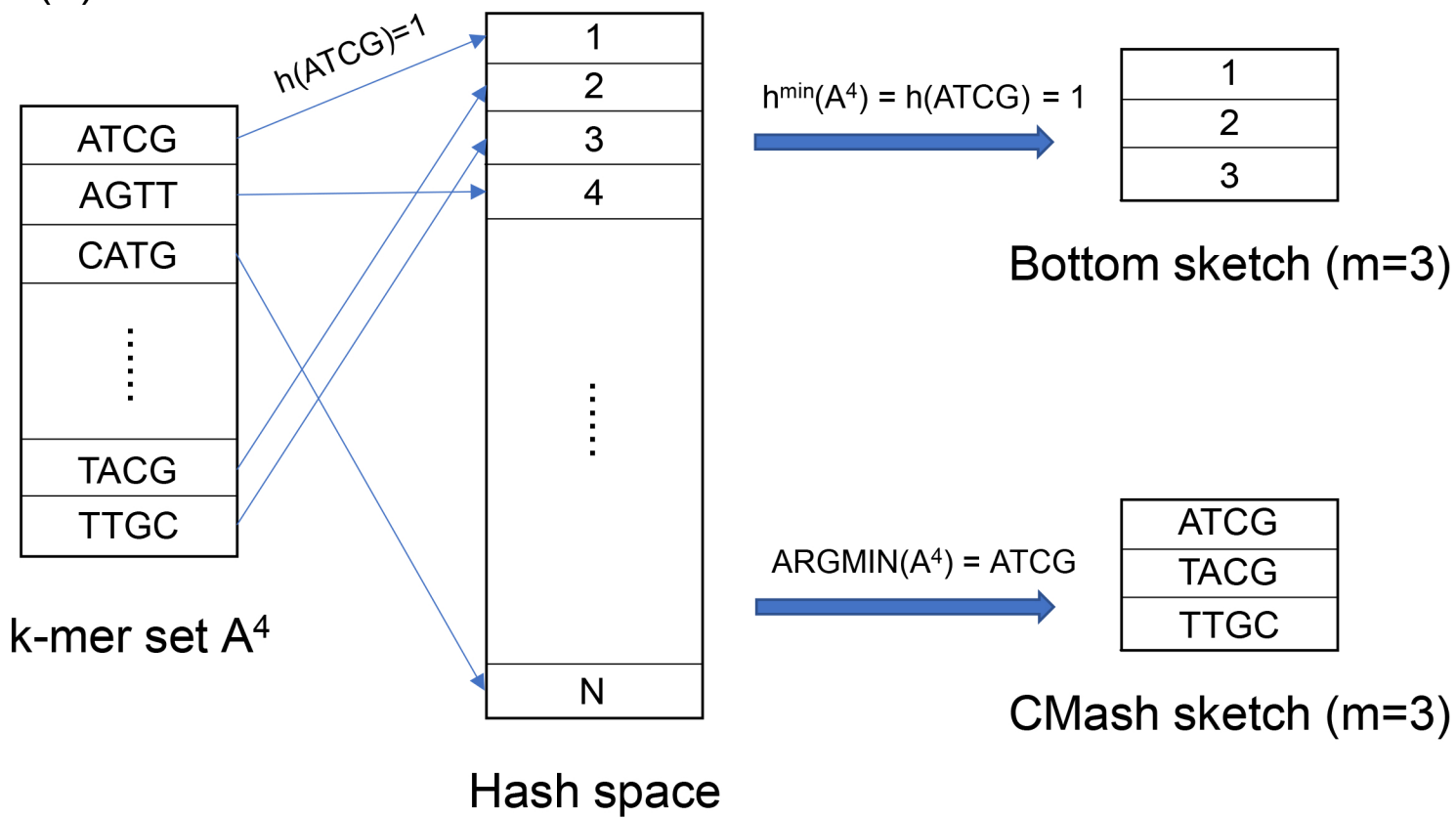

(b)

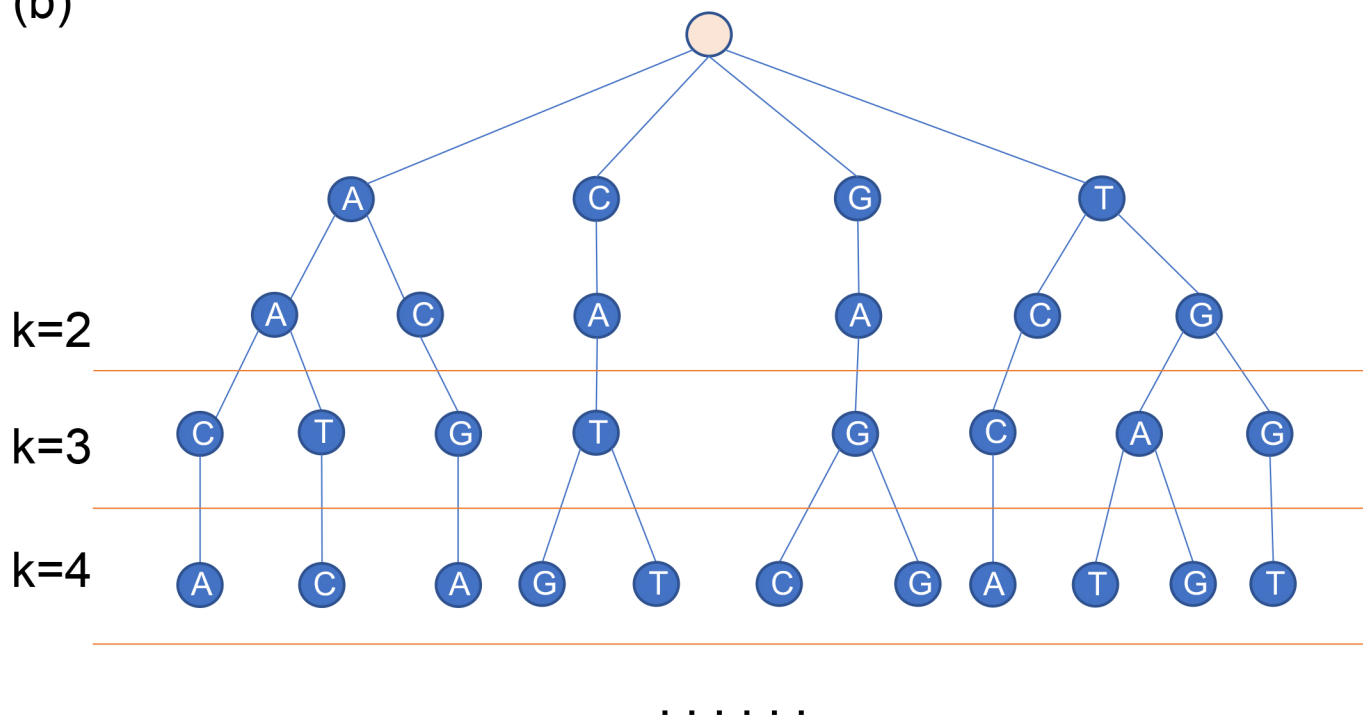

Supplement: btac237_Supplementary_Data [file btac237_supplementary_data.zip › btac237-suppl_data/Koslicki.70.fig.S1.pdf]

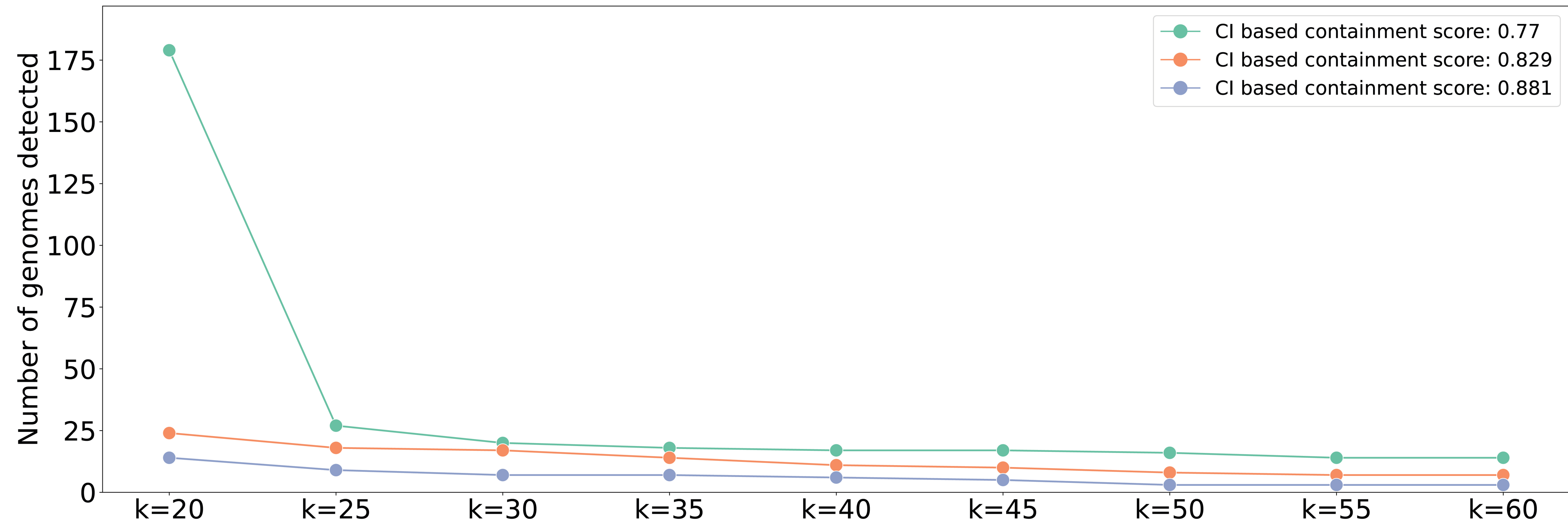

Supplement: btac237_Supplementary_Data [file btac237_supplementary_data.zip › btac237-suppl_data/Koslicki.70.fig.S2.pdf]

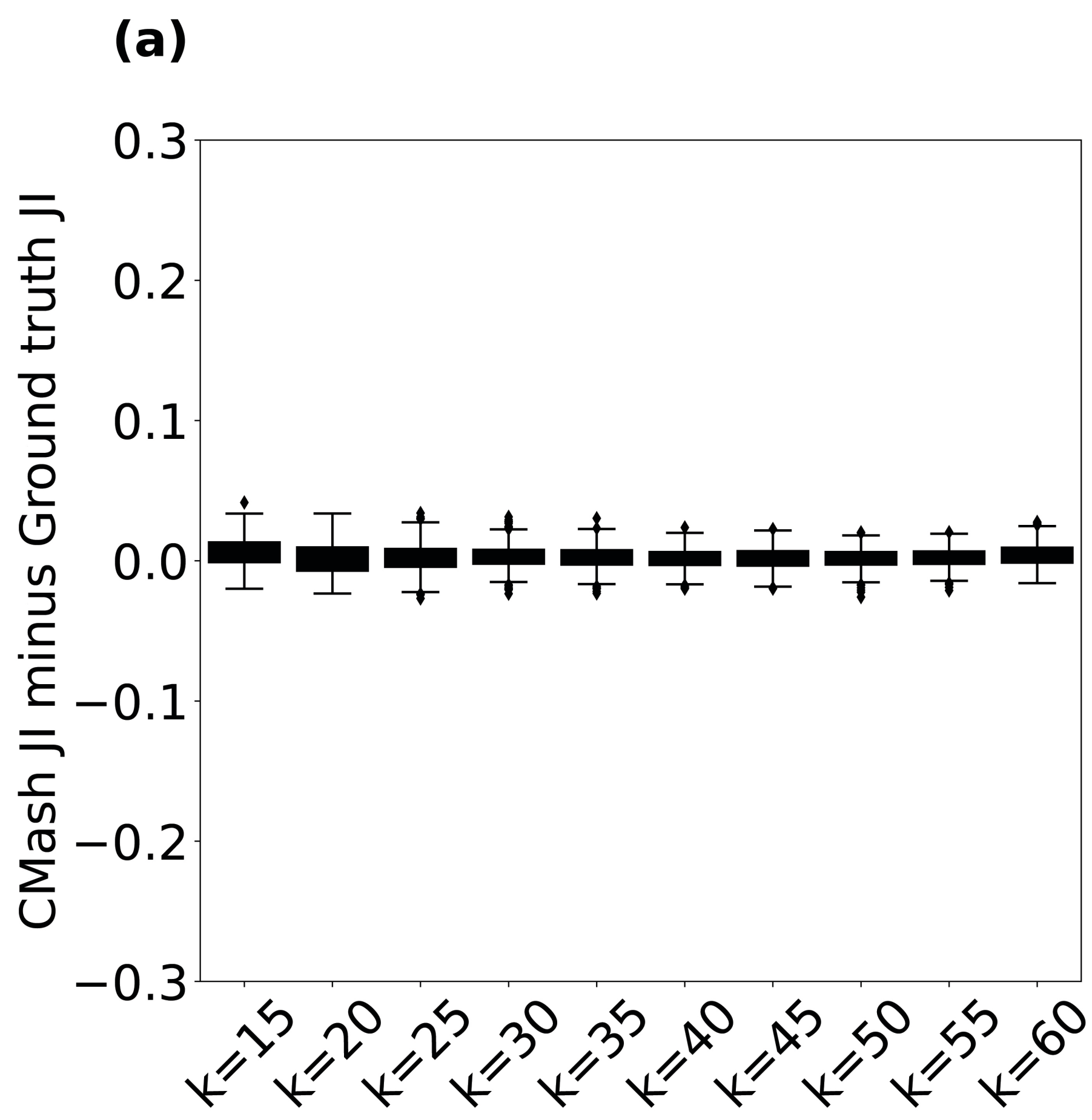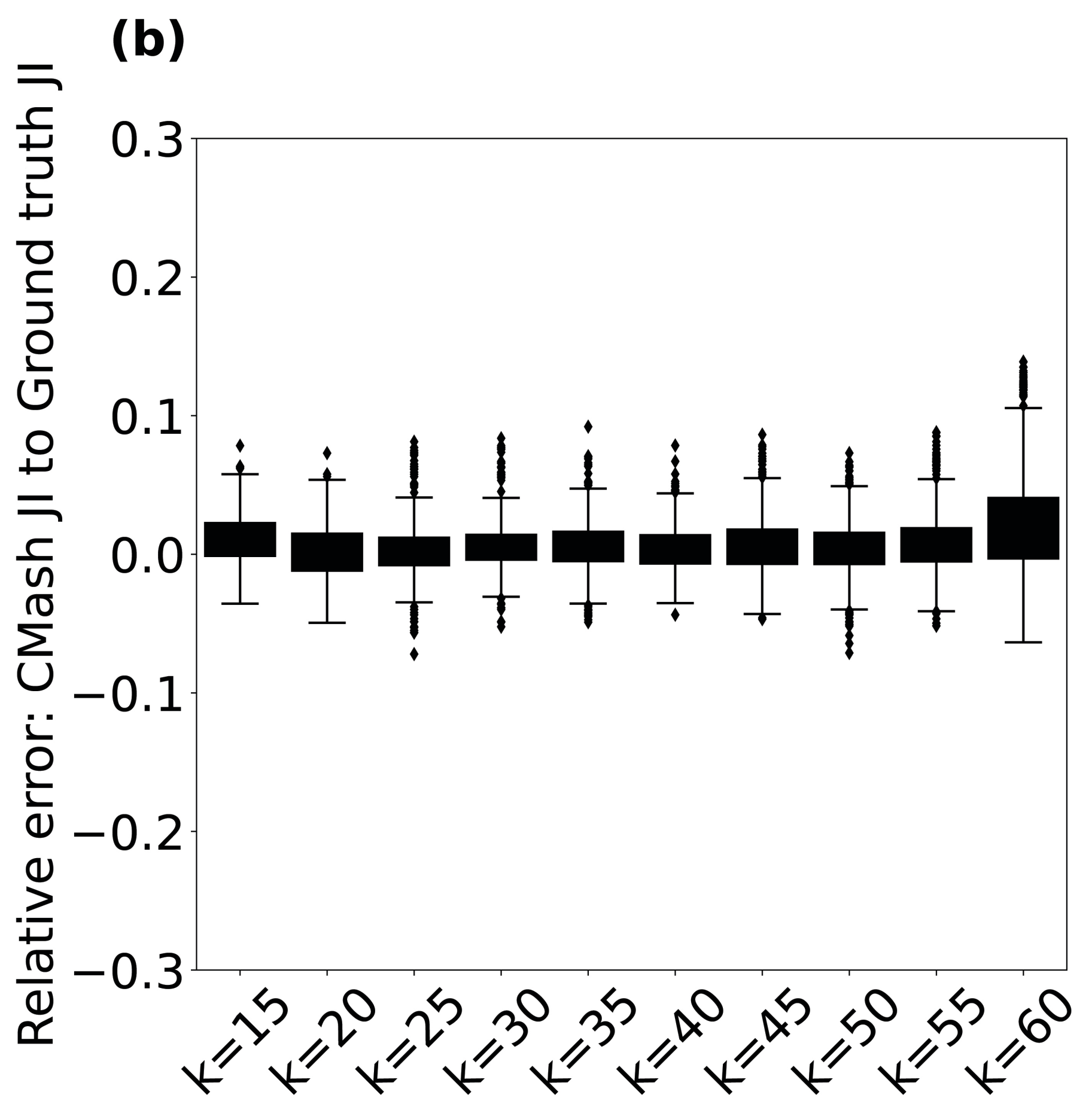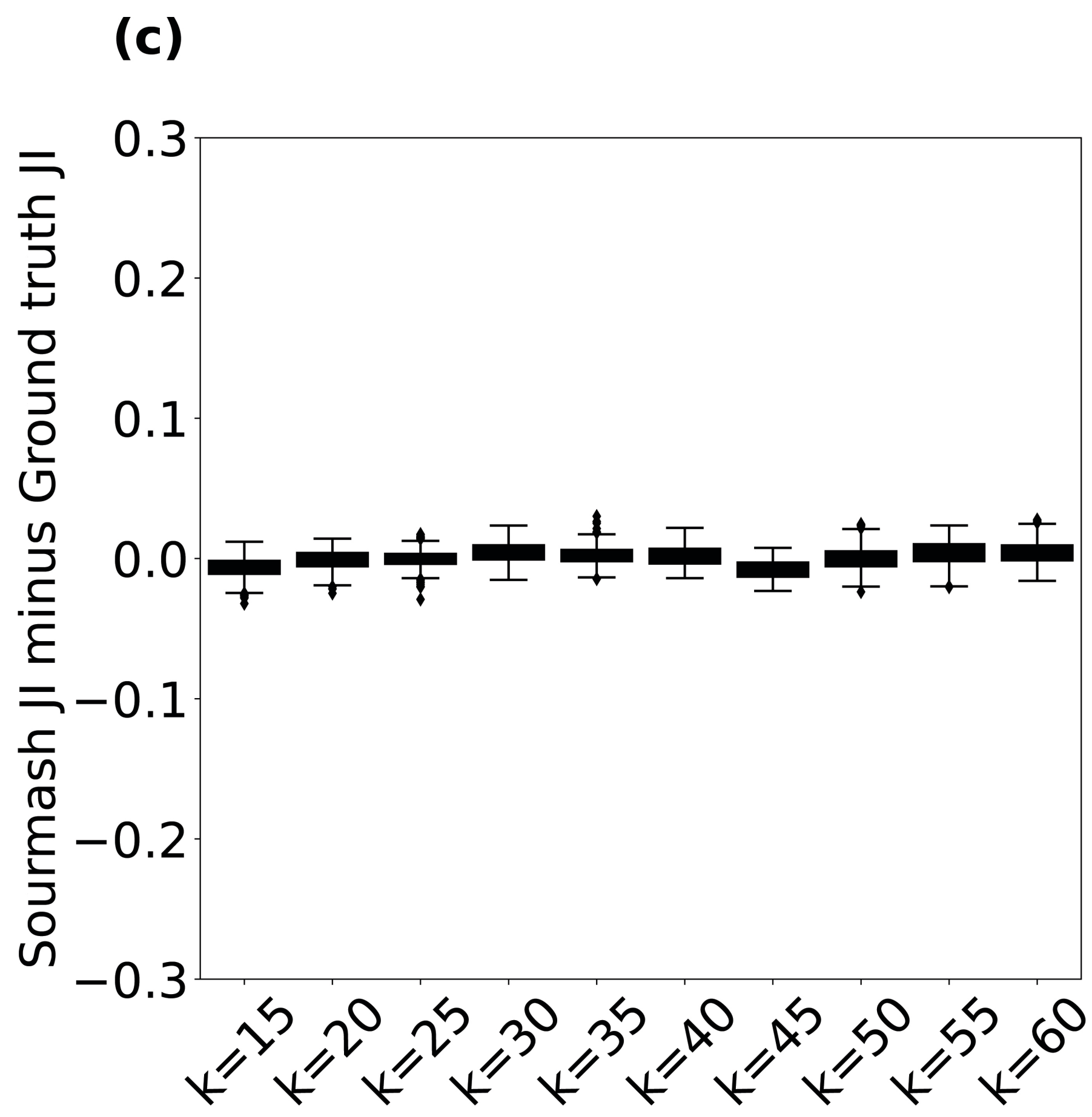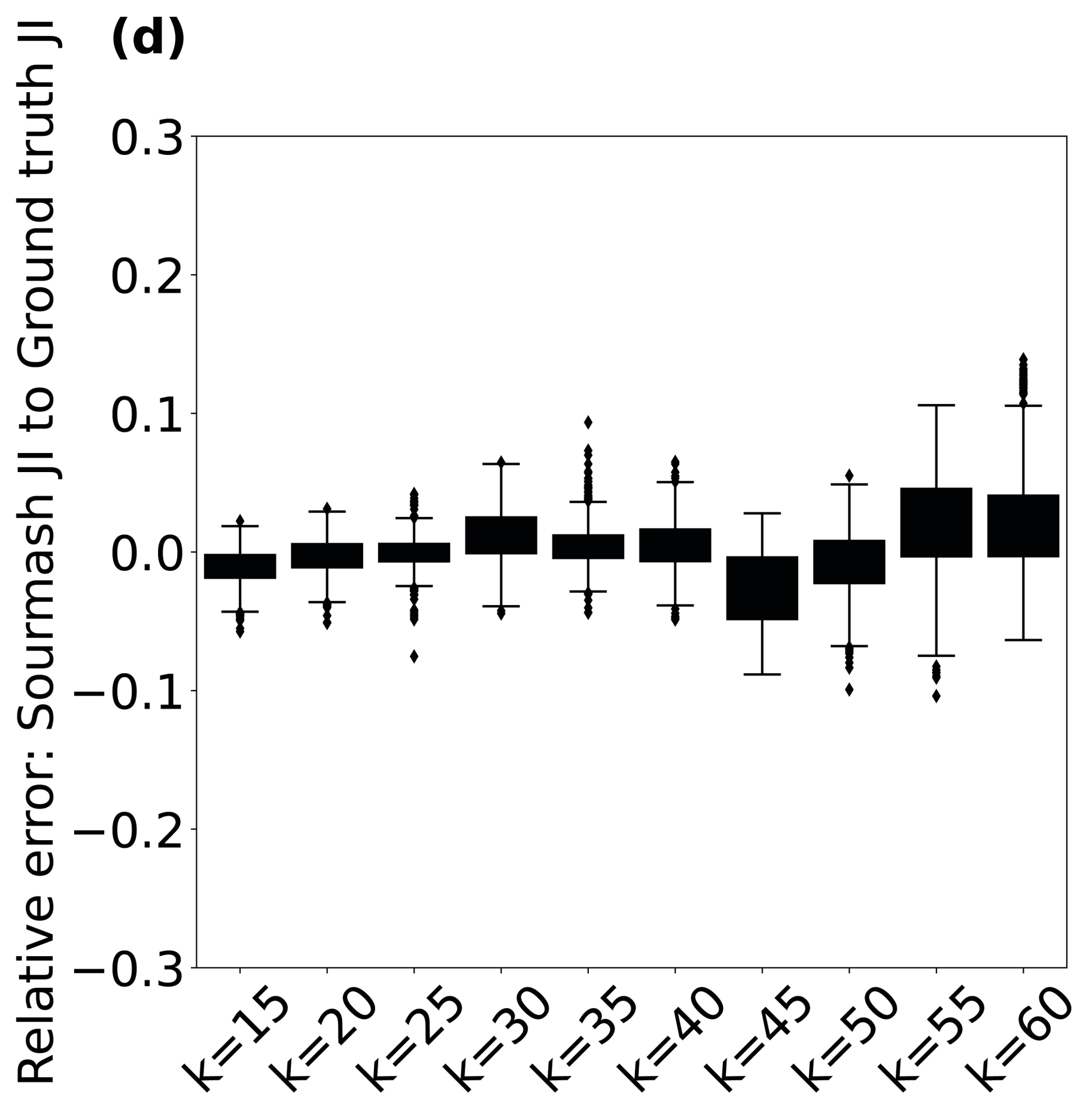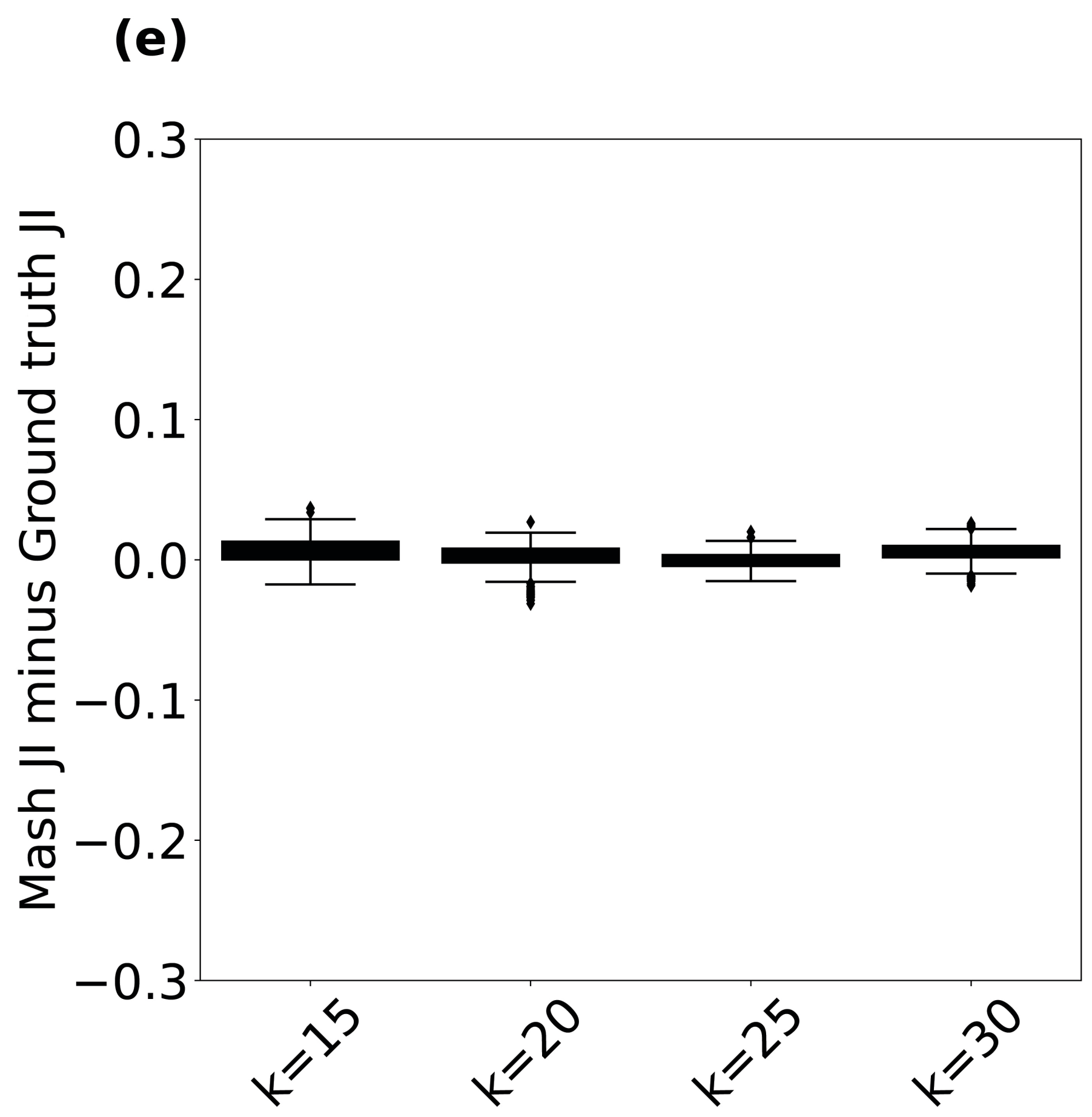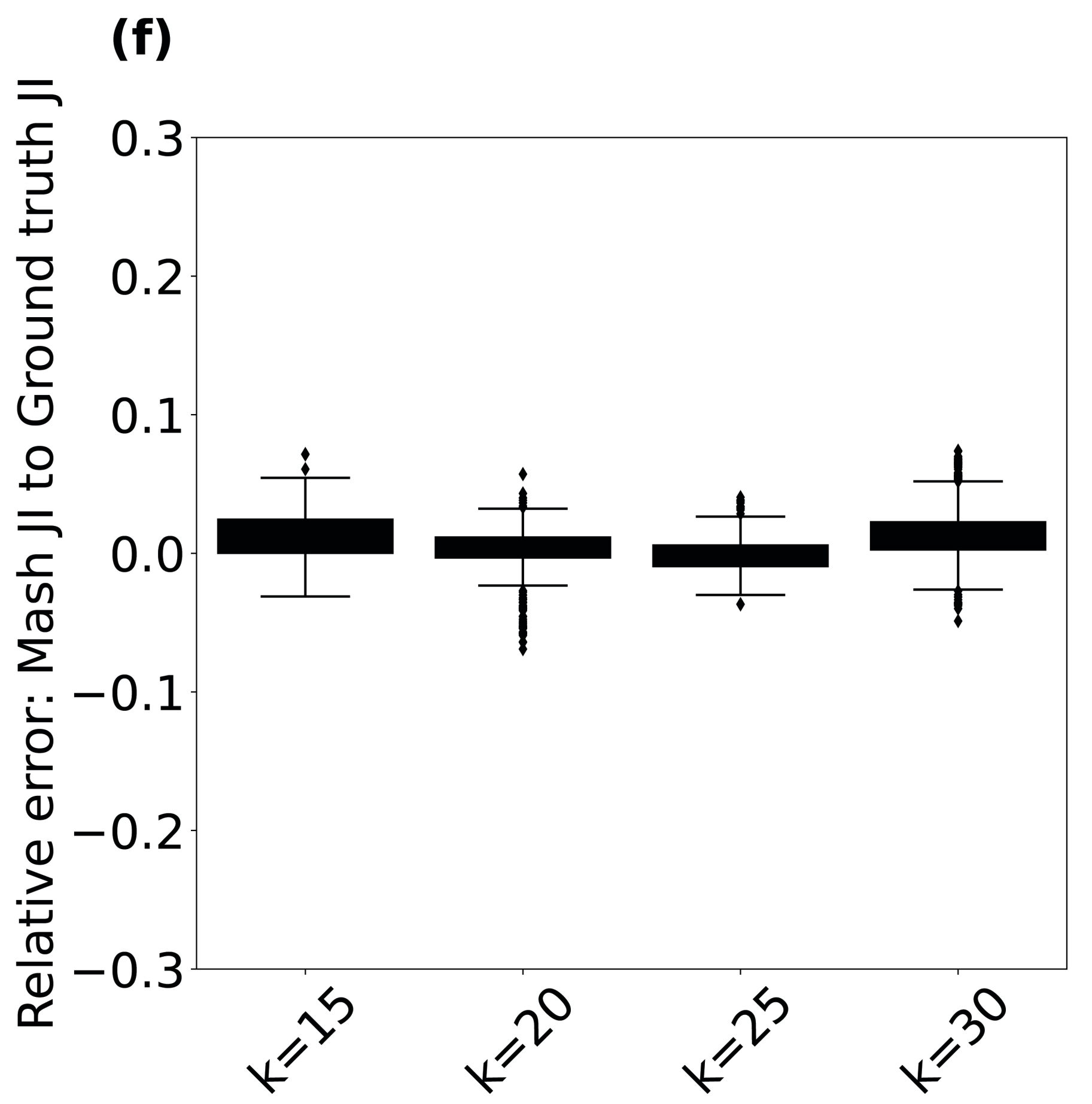

Supplement: btac237_Supplementary_Data [file btac237_supplementary_data.zip › btac237-suppl_data/Koslicki.70.fig.S3.pdf]

**(a)**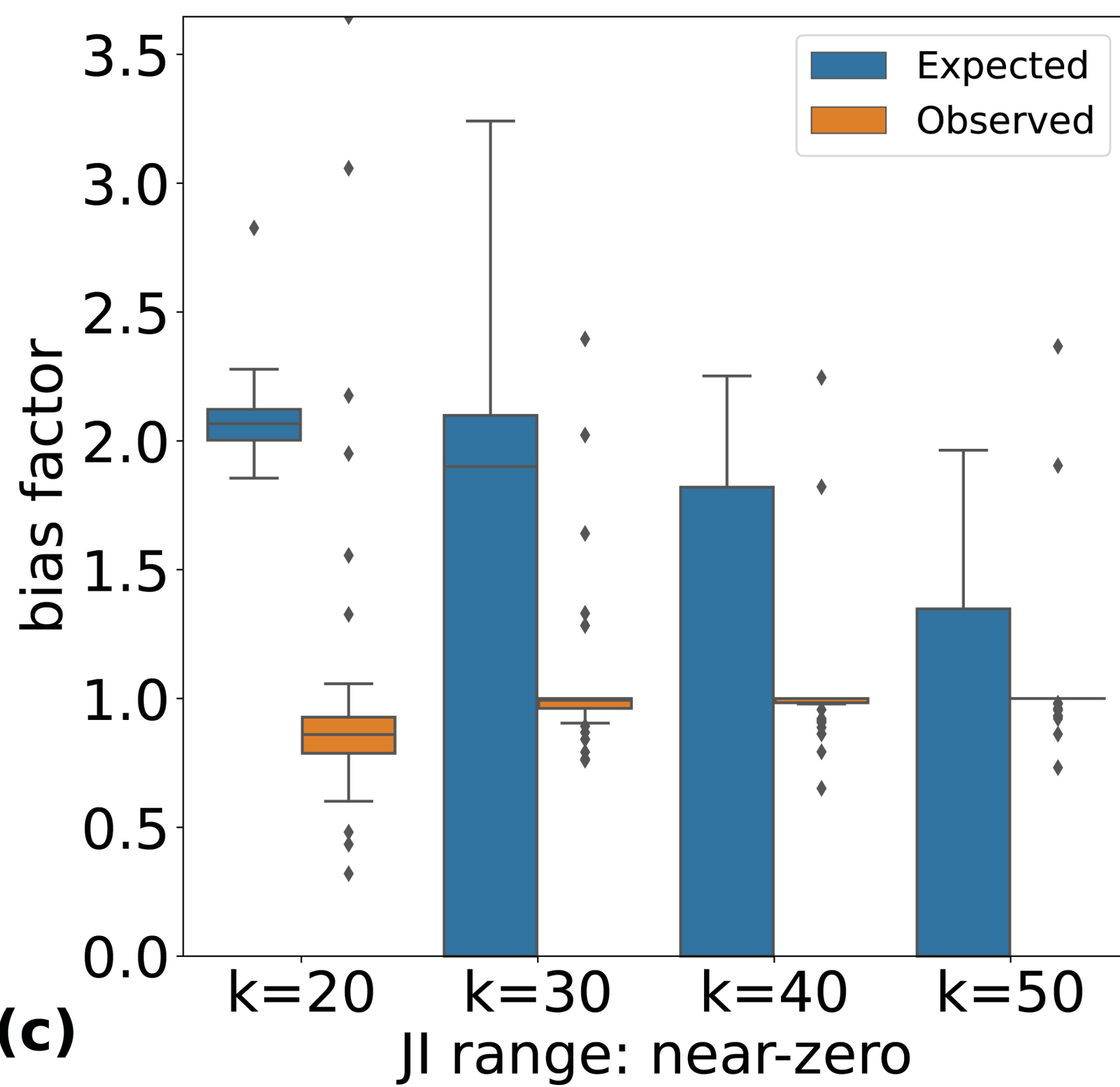**(b)**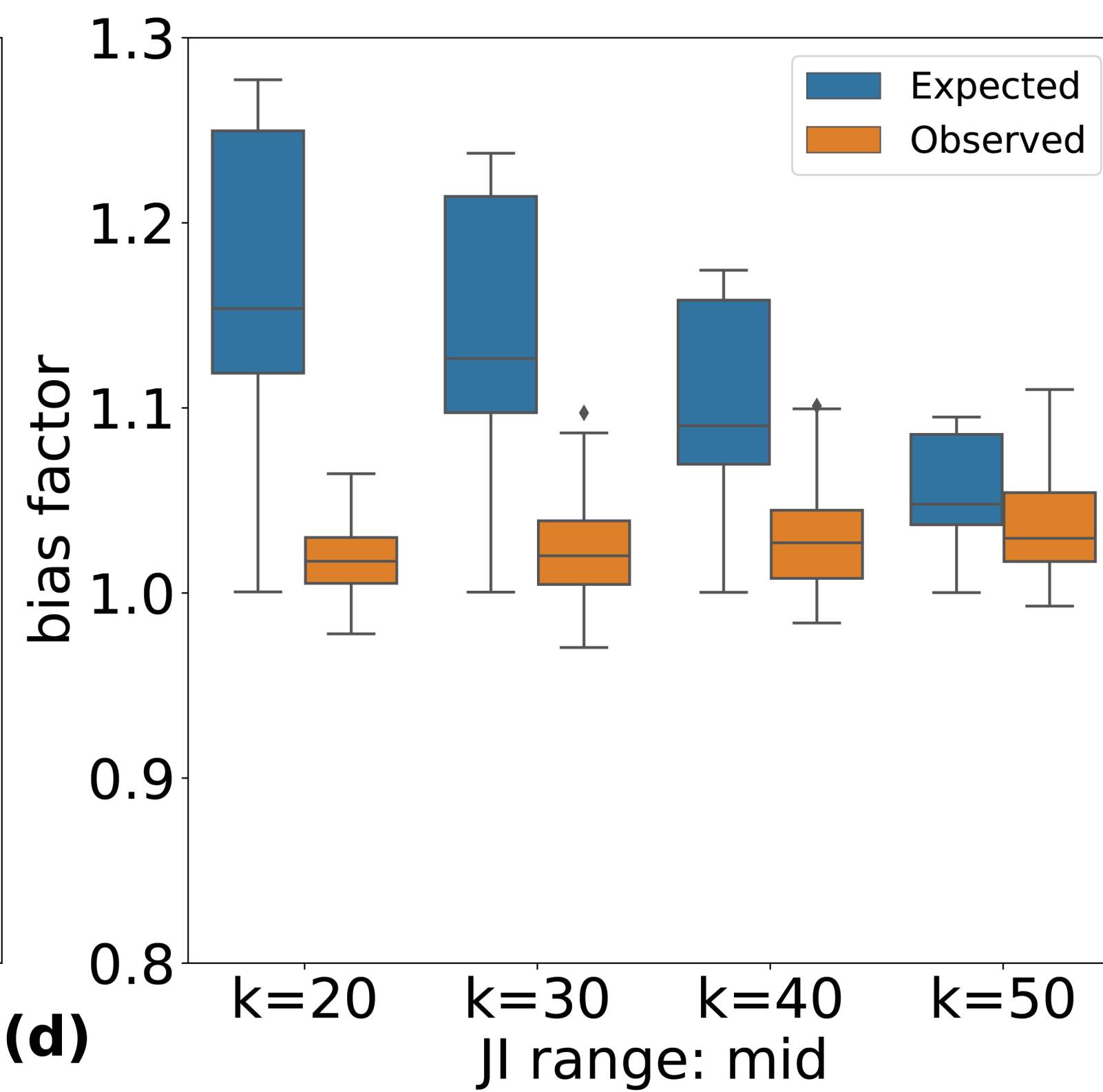**(c)**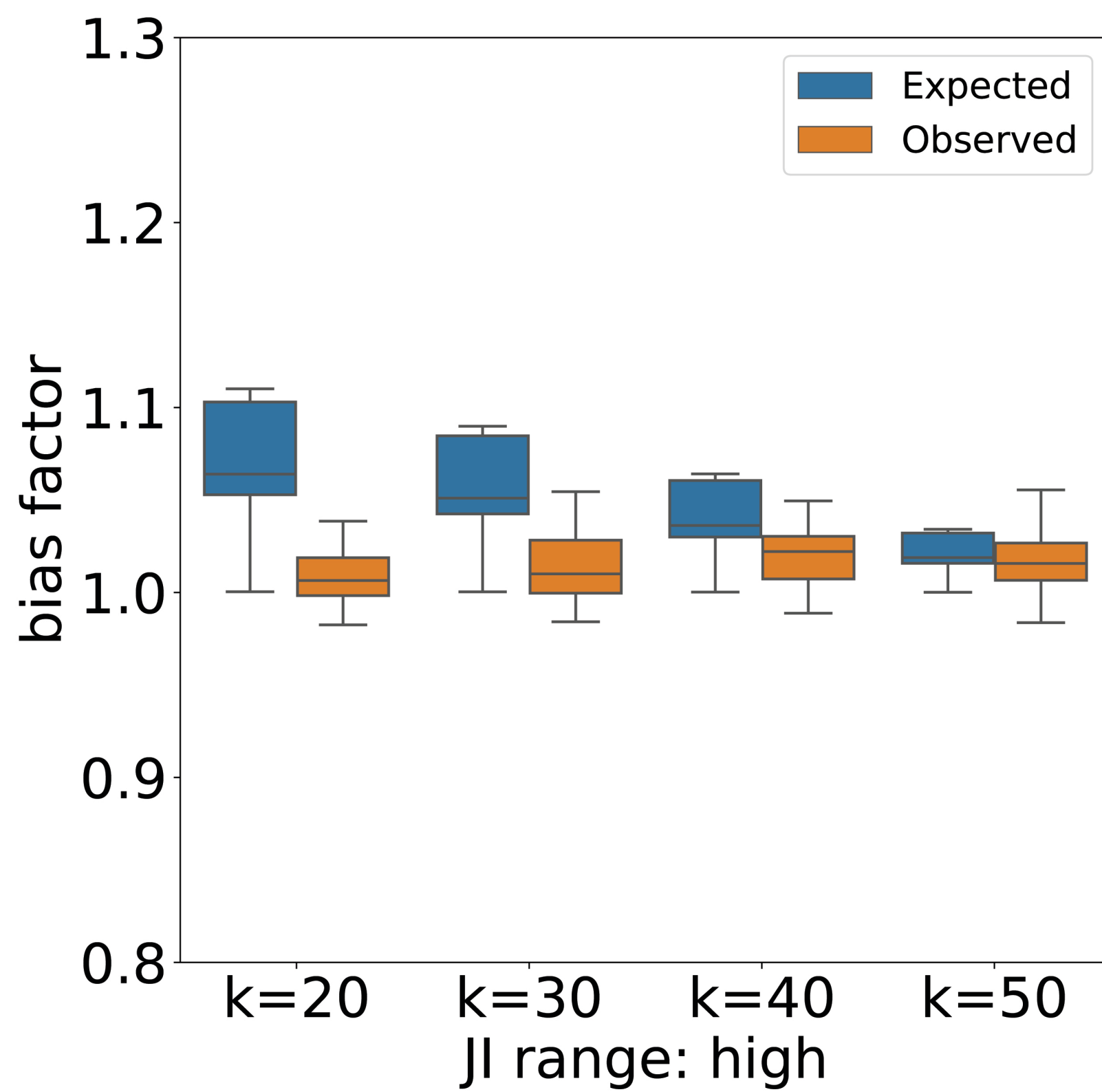**(d)**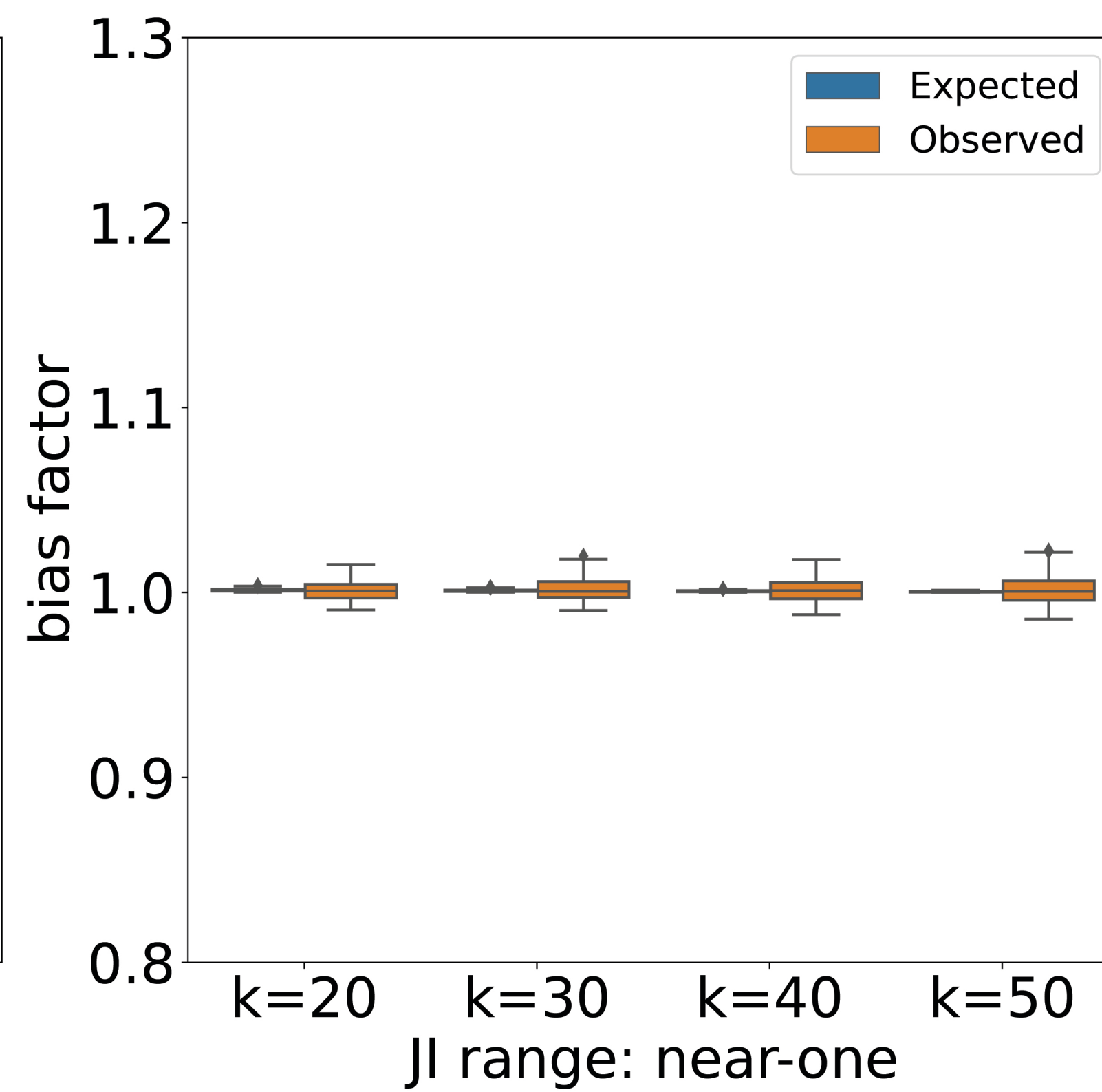

Supplement: btac237_Supplementary_Data [file btac237_supplementary_data.zip › btac237-suppl_data/Koslicki.70.fig.S4.pdf]
